# Magnolia extract is effective for the chemoprevention of oral cancer through its ability to inhibit mitochondrial respiration at complex I

**DOI:** 10.1186/s12964-020-0524-2

**Published:** 2020-04-07

**Authors:** Qi Zhang, Gang Cheng, Jing Pan, Jacek Zielonka, Donghai Xiong, Charles R. Myers, Liang Feng, Song Seok Shin, Young Heui Kim, Dinh Bui, Ming Hu, Brian Bennett, Kathleen Schmainda, Yian Wang, Balaraman Kalyanaraman, Ming You

**Affiliations:** 1 Center for Disease Prevention Research, Medical College of Wisconsin, 8701 Watertown Plank Road, Milwaukee, WI 53226 USA; 2 Department of Pharmacology & Toxicology, Medical College of Wisconsin, 8701 Watertown Plank Road, Milwaukee, WI 53226 USA; 3 Free Radical Research Center, Medical College of Wisconsin, 8701 Watertown Plank Road, Milwaukee, WI 53226 USA; 4 Department of Biophysics, Medical College of Wisconsin, 8701 Watertown Plank Road, Milwaukee, WI 53226 USA; 5 Life Science R&D Center, Osong, Cheongju South Korea; 6 College of Pharmacy, University of Houston, Houston, TX 77204-5037 USA; 7 Department of Physics, Marquette University, Milwaukee, WI 53233 USA

**Keywords:** Oral cancer, AMPK, STAT3, Complex I

## Abstract

**Background:**

Magnolia extract (ME) is known to inhibit cancer growth and metastasis in several cell types in vitro and in animal models. However, there is no detailed study on the preventive efficacy of ME for oral cancer, and the key components in ME and their exact mechanisms of action are not clear. The overall goal of this study is to characterize ME preclinically as a potent oral cancer chemopreventive agent and to determine the key components and their molecular mechanism(s) that underlie its chemopreventive efficacy.

**Methods:**

The antitumor efficacy of ME in oral cancer was investigated in a 4-nitroquinoline-1-oxide (4NQO)-induced mouse model and in two oral cancer orthotopic models. The effects of ME on mitochondrial electron transport chain activity and ROS production in mouse oral tumors was also investigated.

**Results:**

ME did not cause detectable side effects indicating that it is a promising and safe chemopreventive agent for oral cancer. Three major key active compounds in ME (honokiol, magnolol and 4-O-methylhonokiol) contribute to its chemopreventive effects. ME inhibits mitochondrial respiration at complex I of the electron transport chain, oxidizes peroxiredoxins, activates AMPK, and inhibits STAT3 phosphorylation, resulting in inhibition of the growth and proliferation of oral cancer cells.

**Conclusion:**

Our data using highly relevant preclinical oral cancer models, which share histopathological features seen in human oral carcinogenesis, suggest a novel signaling and regulatory role for mitochondria-generated superoxide and hydrogen peroxide in suppressing oral cancer cell proliferation, progression, and metastasis.

Video abstract

## Background

Head and neck cancer refers to a group of biologically similar cancers originating from the upper aerodigestive tract including the lips, oral cavity, nasal cavity, paranasal sinuses, pharynx, and larynx [1]. Head and neck cancers account for about 3–5% of all malignancies in Western countries, with squamous cell carcinoma (SCC) of the oral cavity accounting for 30% of all head and neck cancers [1]. Oral cancer, the sixth most frequent cancer in the world, is the most common malignant neoplasm of the head and neck and a significant global health burden. Most cases have premalignant epithelial lesion stages (e.g. oral leukoplakia and erythroplakia). Tongue cancer accounts for about 40–60% of oral cancer deaths. Despite significant advances in chemotherapy and radiation therapy, five-year survival rates for patients with oral cancer have not changed markedly in the past three decades. Approximately 36,500 new cases are diagnosed, and 8000 patients die annually in the US from this disease.

Magnolia extract (ME) has been known for centuries as a traditional herbal remedy for various disorders such as headache, fever, anxiety, diarrhea, stroke, and asthma [2–6]. ME has various biological effects, including anti-carcinogenic, anti-inflammatory, and anti-anxiety. ME has been used alone and in combination with conventional chemotherapeutics to treat and prevent solid and hematological cancers [5].

Several commercially available ME products contain three major bioactive components: honokiol (HNK), magnolol (MGN), and 4-O-methylhonokiol (MHNK) (Supplemental Table S1) in different proportions. Honokiol (HNK, 5,3′-diallyl-2,4′-dihydroxybiphenyl), a key component in ME preparations [5], has antitumor and anti-metastatic effects in several cell culture and animal models [2, 7, 8]. In tumor cells, HNK suppresses mitochondrial complex I-dependent respiration, stimulates the formation of mitochondrial ROS, induces AMPK activation, and inhibits mitochondrial STAT3 (signal transducer and activator of transcription) phosphorylation [2, 7]. HNK can also affect other signaling pathways including EGFR, Ras/ERK, and PI3K/AKT [9–11]. However, the inhibition of mitochondrial complex I activity and the resulting increases in ROS formation have been proposed to be key aspects of the chemopreventive and antitumor mechanism of HNK. Magnolol (MGN, 5,5′-diallyl-2,2′-dihydroxybiphenyl), another major component in ME [5], significantly inhibits the growth of several human tumor cell lines [12–14] but exhibits no toxicity in normal endothelial cells [15, 16]. MGN can induce tumor cell cycle arrest and mitochondrial apoptosis by regulating the p53 signaling pathway [17, 18]. However, the effects of MGN on mitochondrial complex I activity, ROS formation, AMPK activation, and STAT3 phosphorylation in precancerous or cancer cells are not known. MGN potentiates the cytotoxic effects of HNK in glioblastoma cancer cells [19]. 4-O-Methylhonokiol (MHNK, 3,5′-diallyl-2′-hydroxy-4-methoxybiphenyl) has attracted less attention due to its typically lower abundance in many ME formulations (Supplemental Table S1) [5]. Recent studies suggest that MHNK’s suppression of NF-κB, activation of PPARγ, induction of ROS, disruption of mitochondrial potential and induction of p21 protein expression may lead to antiproliferative effects [20–23] . The effects of MHNK on cancer cell bioenergetics have not yet been investigated.

In this study, we evaluated an ME product (product 7, Supplemental Table S1) that has by far the highest MHNK content as a potent and safe oral cancer preventive agent in a 4NQO-induced mouse oral cancer model and in other oral cancer xenograft models. The effects of ME on mitochondrial electron transport chain activity and ROS production in mouse oral tumors was determined by ex vivo low temperature electron paramagnetic resonance (EPR), biochemical assays of mitochondrial complexes, and the redox state of peroxiredoxins. We demonstrated the potency of ME in preventing oral cancer in mice, with no toxicity observed. A thorough understanding of the mechanism and dose-response effects of the individual components, and multicomponent combinations, is critical to evaluating the antitumor efficacy of ME. Our results suggest that the antiproliferative efficacy of ME results from an inhibition of mitochondrial respiration and resulting redox signaling in oral cancer cells.

## Methods

### Cell lines, reagents and animals

Human oral cancer cell lines SCC-4, SCC-9 and CAL 27 cells were purchased from ATCC where they perform short tandem repeat profiling for cell line authentication. Cells were maintained in RPMI 1640 medium (Gibco, Waltham, MA, USA), supplemented with 10% fetal bovine serum and antibiotics (100 units/ml penicillin and 100 mg/ml streptomycin). Cells were incubated at 37 °C in a humidified atmosphere of 5% CO_2_ in air. Normal human bronchial epithelial cells (NHBE) were purchased from Lonza (Alpharetta, GA) which authenticated the cells by thorough QC testing: NHBE were cultured in bronchial epithelial growth medium (BEGM) at 37 °C in a humidified atmosphere of 5% CO_2_ in air. All cell lines were used before passage 5 after purchasing. ME was provided by SK Bioland (Seoul, Korea). Honokiol and magnolol were purchased from LKT labs (St. Paul, MN); 4-O-methylhonokiol was purchased from 1717 Chemall Corp. (Mundelein, IL).

Six-week-old female athymic nude mice (Crl:NU (NCr)-Foxn1nu were purchased from Charles River Laboratories (Wilmington, MA). Six-week-old C57BL/6 J mice were obtained from Jackson Laboratory. All studies on animals were approved by the Medical College of Wisconsin Institutional Animal Care and Use Committee (approval number: AUA00001807). Animals were housed with wood chip bedding in environmentally controlled, clean-air rooms with a 12-h light-dark cycle and 50% relative humidity.

### Cell proliferation assay

Cells were seeded in 96-well tissue culture plates at 1000–3000 cells per well. Twenty-four hours after seeding, cells were exposed to various concentrations of ME, whereas control cells received fresh medium with an equivalent amount of dimethyl sulfoxide (DMSO) vehicle. Plates were incubated at 37 °C under 5% CO_2_ and monitored using the IncuCyte Live Cell analysis system (Essen Bioscience, Ann Arbor, MI). The IncuCyte™ Live-Cell Imaging Analyzer provides real-time cell confluence data, which were analyzed using the IncuCyte 2011A software. All assays were performed in triplicate or quadruplicate.

To establish SCC-9 and CAL 27 cells that express luciferase, LV-CMV-puromycin-firefly luciferase was transduced into cells according to the manufacturer’s protocol. Briefly, 1 × 10^5^ cells were plated in 6-well plates and, 24 h later, the medium was replaced with transduction medium containing lentivirus that expresses a puromycin luciferase fusion protein and Polybrene (8 μg/ml). Forty-eight hours after transduction, the infected cells were selected with puromycin (2 μg/ml) for 3 days; pooled cells that stably expressed luciferase were used in the study.

### PathScan receptor tyrosine kinase assay

CAL 27 cells were treated with vehicle control (DMSO), HNK (20 μM), MGN (40 μM), and MHNK (20 μM) for 4 h and then lysed with a lysis buffer containing a proteinase inhibitor cocktail (Cell Signaling Technology, Danvers, MA), sheared 10 times by passage through a 28-gauge needle, and centrifuged at 16,000 *g* for 30 min; the protein concentration of the supernatant was determined by the Bradford method [24]. Lysates, normalized for equal protein loading, were analyzed by the PathScan RTK Signaling Array (Cell Signaling Technology, Danvers, MA) and the LI-COR Odyssey infrared imaging system (LI-COR Biosciences-Biotechnology, Lincoln, NE).

### Western blot analyses

Cells were lysed with RIPA buffer containing a proteinase inhibitor cocktail (Fisher Scientific, Pittsburg, PA), sheared 10 times by passage through a 28-gauge needle, and centrifuged at 16,000 *g* for 30 min; the supernatants were normalized for protein concentration as determined by the Bradford method. Lysates were boiled for 5 min and resolved on 4–12% sodium dodecyl sulfate polyacrylamide gel electrophoresis (SDS-PAGE) gels (Invitrogen, Carlsbad, CA). The blots were probed with the following primary antibodies from Cell Signaling Technology (Danvers, MA): p-STAT3-Tyr705, p-STAT3-Ser727, HKII, P70, and P70S6K. The following antibodies were from Santa Cruz Biotechnology (Dallas, TX): anti-actin, anti-Prx1 and anti-Prx3.

### Respiratory enzyme activity

Mitochondrial function was measured using a Seahorse XF96 Extracellular Flux Analyzer (Seahorse Bioscience, North Billerica, MA), as described previously [25–28]. For measurement of mitochondrial respiratory complexes, after 24-h treatments as indicated, intact cells were permeabilized using 1 nM Plasma Membrane Permeabilizer (PMP, Seahorse Bioscience) immediately before OCR measurement. The oxygen consumption derived from mitochondrial complex I or II activity was measured by providing different substrates to mitochondria, e.g., pyruvate/malate for complex I and succinate for complex II. Rotenone, malonate, and antimycin A were used as specific inhibitors of mitochondrial complex I, II, and III, respectively.

### Redox blots for peroxiredoxins

Redox western blots for Prx1 and Prx3 were done as previously described [29, 30]. Following treatment with ME or vehicle control, cells were harvested to capture the protein thiol redox state: cells were washed once with Hank’s Balanced Salt Solution (Life Technologies, Carlsbad, CA) and immediately scraped into *N*-ethylmaleimide (NEM) extract buffer (40 mM HEPES pH 7.4, 50 mM NaCl, 100 mM NEM, 1 mM EDTA, 1 mM EGTA, 1 mM PMSF, 10 μg/mL catalase). After 15 min at room temperature, the cells were pelleted (5 min, 800 *g*), and then lysed on ice in 35 μL NEM extract buffer containing 1% CHAPS (3-[(3-cholamidopropyl) dimethylammonio]-1-propanesulfonate). The lysates were held at − 80 °C until analysis. Lysates were thawed on ice and centrifuged for 5 min (8000 *g*, 4 °C). The supernatants were run on non-reducing SDS-PAGE (NuPAGE Novex 10% Bis-Tris gels), and the blots were probed with anti-Prx1 or anti-Prx3, followed by the appropriate HRP-conjugated secondary antibody. Multiple exposure times were captured. The percent of oxidized vs. reduced Prx was determined by image densitometry using UN-SCAN-IT software v.6.1 (Silk Scientific, Orem, UT).

### Ex vivo measurement of mitochondrial complex activity in tumor tissues

The effect of ME on mitochondrial ETC complexes in mouse oral cancer was determined by ex vivo low-temperature EPR. We have previously used this approach to measure changes in mitochondrial complexes in cell culture systems in vitro and tissues ex vivo [31, 32]. Briefly, oral tumors from control and ME-treated animals were flash frozen in liquid nitrogen, minced and transferred to EPR tubes. EPR measurements were performed at liquid helium temperatures (10–30 K).

### Efficacy of ME on SCC-9 orthotopic oral cancer in nude mice

Six-week-old female athymic nude mice (Crl:NU (NCr)-Foxn1nu, Charles River) were used to evaluate the effect of ME. After anesthesia using isoflurane, 1 × 10^5^ of SCC-9-luc cells in 30 μL PBS were injected into the lateral portion of the tongue. Mice were imaged for firefly luciferase expression (150 μg/g body weight D-luciferin) using an IVIS 100 Xenogen monitor (Xenogen, Alameda, CA) imaging system at multiple timepoints post injection. Seven days after engrafting SCC-9 cells, ME (120 mg/kg body weight) was orally administered for 6 weeks, 5 times per week. Mice were sacrificed at the endpoint.

### Efficacy of ME on oral carcinogenesis

The 4NQO-induced mouse oral cancer model has been commonly used to assess the effect of natural compounds on oral carcinogenesis [33–35]. Briefly, 6-week-old C57BL/6 J mice were randomized into 5 groups: 1) control; 2) HNK (20 mg/kg body weight); 3) MGN (20 mg/kg body weight); 4) MHNK (20 mg/kg body weight); 5) 120 mg/kg body weight ME (Product 7, Supplemental Figure S1). Oral tumors were induced by exposing mice to 4NQO at a dose of 50 μg/mL in drinking water for 16 consecutive weeks. One week following the first dose of 4NQO, mice were given ME or the individual active components in ME via oral administration (five days/week for the next 20 consecutive weeks). Animal body weights and 4NQO-water consumption were monitored weekly. 21 weeks after the start of 4NQO treatment, mice were imaged by MRI. Mice were euthanized and oral tissues were collected to assess total oral tumor volume (sum of V) for each mouse. A portion of normal appearing tissue and oral tumor from each mouse were flash frozen in liquid nitrogen for molecular analysis. Remaining oral tissues were embedded in paraffin for pathologic analysis as previously described and for immunohistochemistry (IHC) analysis of biomarkers. In addition, the oral cavity, esophagus, stomach/forestomach, lung, and other major organs were examined carefully for any signs of abnormality.

### Magnetic resonance imaging (MRI)

Mice were imaged using a 9.4 T MRI (Bruker, Billerica, MA) with a custom birdcage style Quadrature coil (Doty Scientific, Columbia, SC). Mice were anesthetized (2 min) initially in a chamber using a mixture of oxygen (3 L/min) and isofluorane (4%). Once an animal was fully anesthetized, optical lubricant was placed on each eye to prevent drying. The mouse was then moved to the MRI magnet room and placed in a supine position on a plastic bed fitted with a nose cone that continuously supplied oxygen (1.4 L/min) and isofluorane (1.5–2.0%). Tumors were imaged using a multi-slice, multi-echo acquisition (MSME). Images were acquired using the following parameters; T2-weighted: T2 Turbo RARE (flip = 90^o^; TE/TR = 42/2500 ms; matrix = 192 × 192; slice = 0.75 mm).

### RNA-sequencing (RNA-seq) assays

RNA-seq experiments were conducted on the oral tumor samples extracted from ME-treated and non-treated mice (*n* = 3 per group). Total RNA was extracted using the Qiagen RNeasy Mini Kit. We used the NEBNext Ultra RNA Library Prep Kit to construct the RNA-seq libraries, which were then sequenced using the Illumina HiSeq 2500 platform. Approximately 185 million 50-bp single-end RNA-seq reads were generated, with an average of over 30 million sequence reads per sample. The read alignments and annotation were conducted using Bowtie-TopHat. Read counts were obtained using HTseq. Data normalization and differential expression analysis were performed using the statistical algorithms implemented in EdgeR Bioconductor package [36]. FDR, i.e., corrected *P* values of less than 0.05, was used as the criterion for identifying significantly regulated genes. Pathway analysis was conducted using IPA software (Qiagen, MD).

### Pharmacokinetics of ME

The three key components of ME (HNK, MGN, MHNK) were evaluated in animals using LC-MS/MS analysis. C57BL/6 J female mice weighing approximately 20 g each were randomly assigned into five groups. Animals were given ME (120 mg/kg) daily for 1 week and sacrificed by CO_2_ asphyxiation at 0, 2.0, 4.0, 6.0, and 24 h after the last treatment. Oral cavity, blood and tongue samples were collected. Blood samples from the retro-orbital plexus of each animal were collected in EDTA-treated tubes. The blood sample (20 µL) was spiked with 20 µL of 80% MeOH and 160 µL of 100 nM internal standard (lS - Baohuoside I) in ethyl acetate. The mixture was vortexed for 1 min. After centrifugation at 15,000 rpm for 15 min, the supernatant was transferred to a new tube and evaporated to dryness under a stream of airflow. The residue was reconstituted in 140 µL of 80% methanol and centrifuged at 15,000 rpm for 15 min. 10 µL of supernatant was injected into the UPLC-MS/MS system for quantitative analysis [37]. The oral cavity and tongue tissue samples (about 20 mg each) were homogenized by Storm BBY24M bead mill homogenizer (Next Advantage, N.Y. USA) in 500 μL saline. The homogenized mixture was processed with the same extraction procedure of blood samples.

### Statistical analysis

GraphPad Prism software was used for evaluating statistical differences between treatments. Student’s t-test was applied for pairwise comparisons. For assessing multiple comparisons (e.g., inhibition of viability data) we used ANOVA with Tukey’s post-hoc test. *p*-Values < 0.05 were considered significant.

## Results

### ME and ME components inhibits oral cancer cell proliferation

**Fig. 1:**
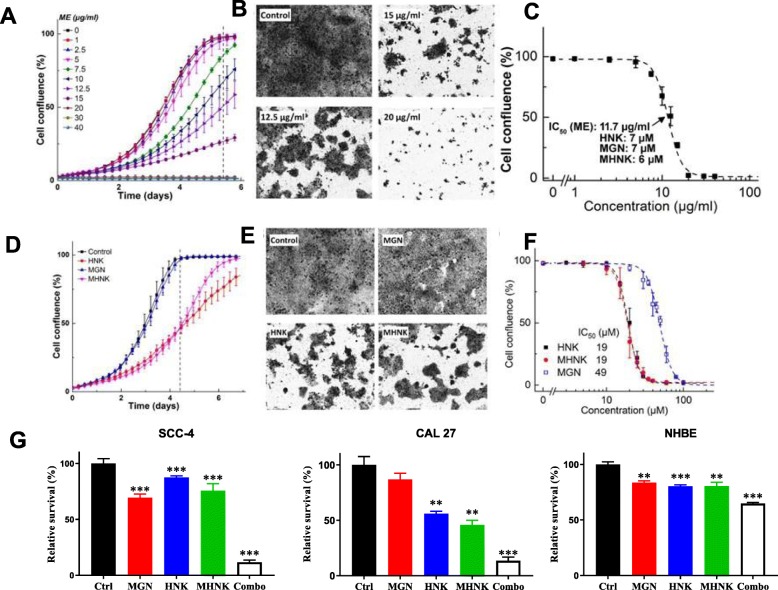
Effects of ME, HNK, MGN and MHNK on human oral cancer cells and normal human bronchial epithelial (NHBE) cells proliferation. **a** Representative cell growth curves of CAL 27 upon treatment with various doses of ME. **b** Cell images corresponding to panel A, at the time point indicated by the vertical dashed line. **c** Dose-response effects of ME on cell confluence. The indicated concentrations of HNK, MGN, and MHNK are those contained in the 11.7 μg/ml ME treatment. **d** Representative cell growth curves upon treatment with vehicle or 20 μM of each compound. **e** Cell images corresponding to panel **d**, at the time point indicated by the vertical dashed line. **f** Dose-response antiproliferative effects of each of the three active compounds found within ME. **g** Effects of HNK, MGN and MHNK (20 μM each) and the mixture of all three agents (Combo, 20 μM each) on the proliferation of two human oral cancer cell lines (SCC4 and CAL27) and normal human bronchial epithelial (NHBE) cells

The efficacy of ME and ME’s main components (HNK, MGN and MHNK) on the survival of the CAL 27 oral cancer cell line was tested. Real-time cell confluence data from the IncuCyte Analyzer show that ME, HNK, MGN and MHNK inhibit human oral cancer CAL 27 cell growth. The IC_50_ for ME is 11 μg/mL, which contains 7 μM HNK, 7 μM MGN, and 6 μM MHNK (Fig. 1a-c). The IC_50_ values for HNK, MGN, and MHNK when used as single agents are 19, 49 and 19 μM, respectively (Fig. 1d-f). A synthetic mixture of HNK, MGN and MHNK, at the same ratio as in ME, yielded the same IC_50_ value as was obtained with ME, indicating that these three compounds (and not other components) are responsible for ME’s antiproliferative effects. These concentrations of the active agents in ME (Fig. 1a-c) are much lower than the IC_50_ values for HNK, MGN and MHNK as individual components (Fig. 1d-f). This implies a potential synergy between these ME components, which suggests that there may be different specific cellular targets for each ME bioactive component and/or different anti-proliferative mechanisms.

Next, the efficacy of each compound individually (HNK, MGN, MHNK, 20 μM each) versus the mixture of all three (20 μM each) on the survival of two oral SCC cell lines (SCC-4 & CAL 27) and non-cancer normal epithelial cells (NHBE) was tested. The mixture was markedly more effective against cancer cells than any of the individual agents, but only had minor effects on the non-tumorigenic NHBE cells (Fig. 1g). These data show that ME’s components markedly suppress the survival of oral cancer cells but have only minor effects on normal cells even at the high concentration (60 μM total) in the mixture.

### ME inhibits mitochondrial complex I-mediated respiration

**Fig. 2:**
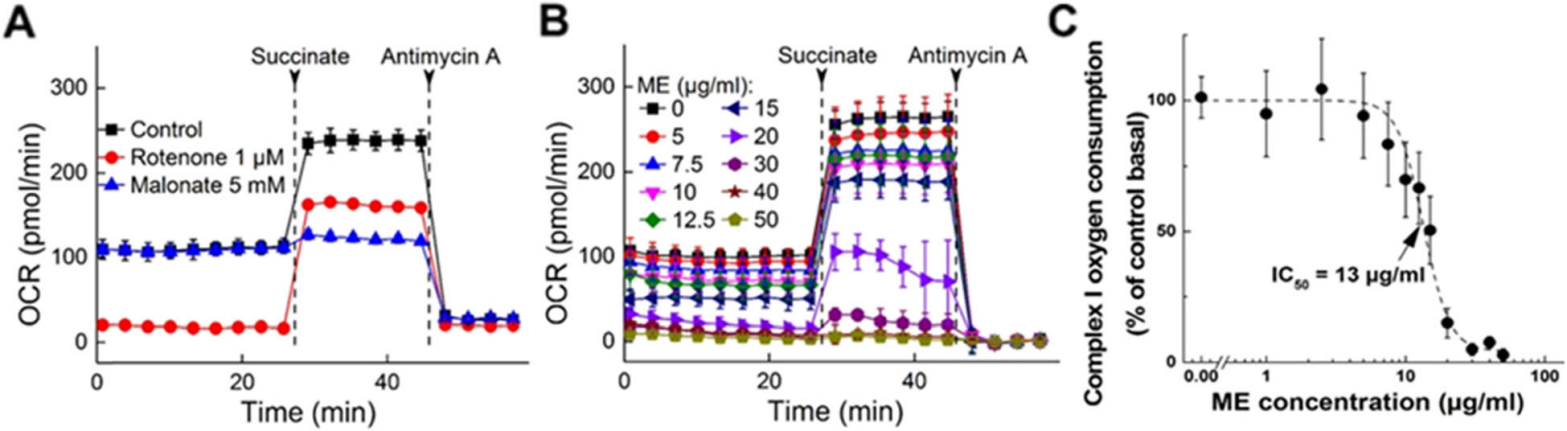
Effect of ME on mitochondrial complex I activity in CAL 27 cells. **a** Experimental model setup for complex I and II activity measurements. **b** OCR traces recorded, and **c** complex I activity as a function of ME concentration

To assess mitochondrial effects of ME, we used a Seahorse XF96 Analyzer that measures in real time OCR, a measure of mitochondrial respiration, and ECAR, a surrogate marker for glycolysis. To test mitochondrial complex activities, we first established the optimal use of permeabilized cells for this purpose (Fig. 2a). OCR is measured after adding substrates and inhibitors of complexes I–IV. Rotenone (complex I inhibitor) diminished OCR that was restored by added succinate (complex II substrate), while in the presence of malonate (complex II inhibitor) succinate did not stimulate OCR. Antimycin A (complex III inhibitor) decreased both pyruvate- and succinate-induced OCR. ME, added 10 min before the first reading, rapidly inhibited complex I activity in human oral cancer CAL 27 cells (Fig. 2b). The IC_50_ for ME to inhibit complex I-mediated respiration was 13 μg/ml (Fig. 2c), close to its IC_50_ (11.7 μg/ml) for cell proliferation (Fig. 1c). This rapid inhibition suggests that mitochondrial complex I is a primary proximal target of ME, and that the inhibition of complex I is a key initial event that is responsible for the subsequent anti-proliferative effects of ME against human oral cancer cells.

### ME induces Prx oxidation in oral cancer cells

**Fig. 3:**
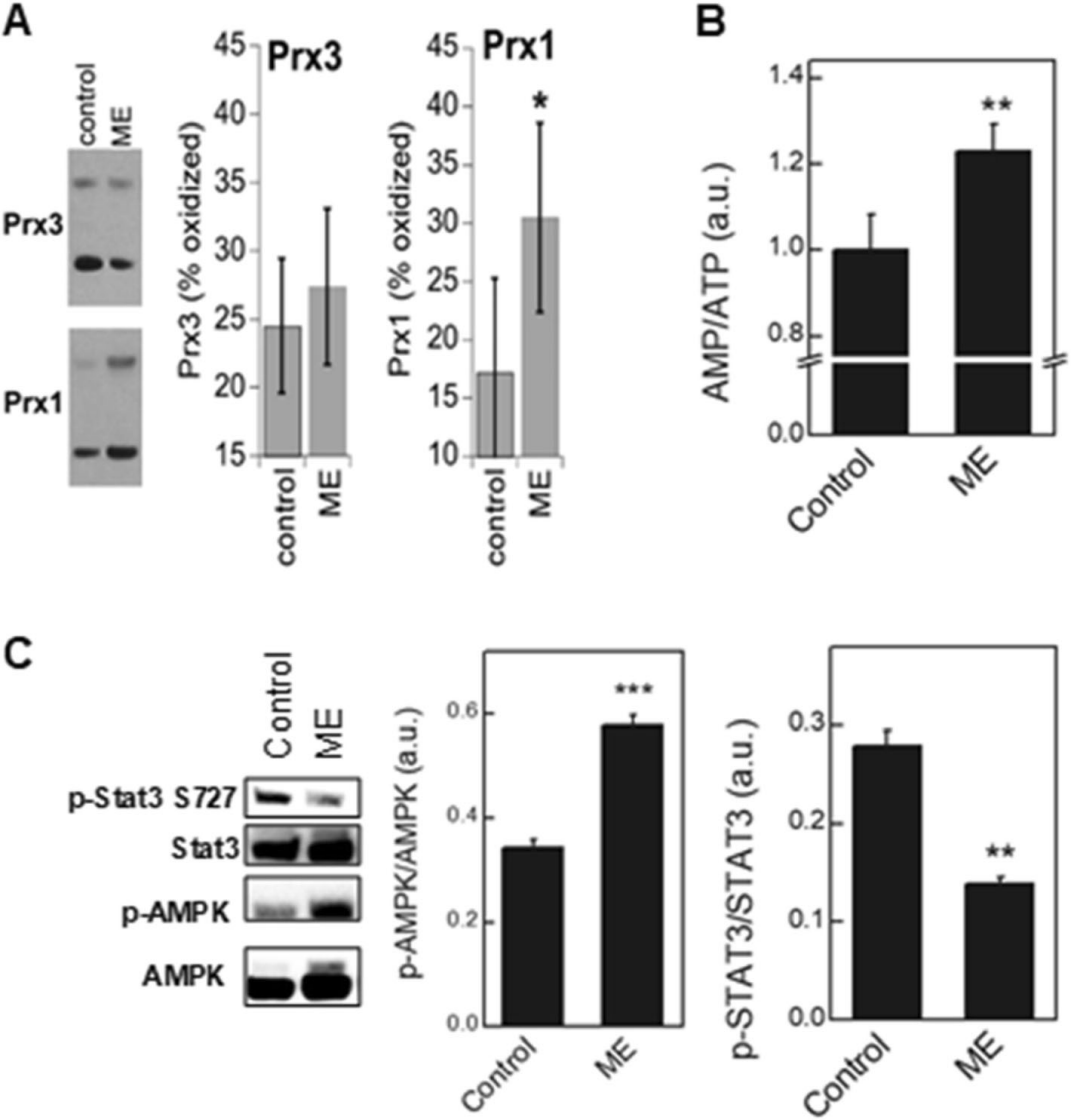
Effect of ME on the redox status, cellular bioenergetic status and phosphorylation of AMPK and STAT3 in CAL 27 cells. **a** redox western blots for oxidized and reduced mitochondrial (Prx3) and cytosolic (Prx1) peroxiredoxins; **b** AMP/ATP ratio, as measured by LC-MS; **c** Representative western blots and image densitometry data (bar graphs) for total AMPK and STAT3 and their phosphorylated forms (p-AMPK and p-STAT3^S727^). *p < 0.05; **p < 0.01; ***p < 0.001 versus control (vehicle-treated cells)

Inhibition of complex I is known to promote the generation of reactive oxygen species (ROS). Peroxiredoxins (Prxs) are thiol peroxidases that are important in peroxide defense and redox signaling [38]. Due to their high abundance and reactivity, Prxs are primary targets of H_2_O_2_ and are useful compartment-specific redox sensors. ME enhanced the oxidation of cytosolic Prx1 (Fig. 3a) in CAL 27 cells, which implies enhanced cellular peroxide generation. Such peroxide increases may lead to the transfer of the oxidative equivalents to STAT3 or other proteins [39], initiating redox signaling events that result in the inhibition of cancer cell proliferation. While mitochondrial Prx3 also showed a trend toward greater oxidation (Fig. 3a), it was not statistically significant.

### Bioenergetic status and AMPK activation

Mitochondrial inhibition would be expected to result in changes in bioenergetics status. We therefore tested the effects of ME on the intracellular AMP/ATP ratio using LC-MS/MS-based analyses, and on AMPK activation by monitoring its phosphorylation status by Western blotting. ME increased the AMP/ATP ratio (Fig. 3b) accompanied by activation (phosphorylation) of AMPK (Fig. 3c). Increased ROS, including H_2_O_2_, are an alternative mechanism for activating AMPK that does not require an increased AMP/ATP ratio. ME led to decreased levels of p-STAT3^S727^ and increased the phosphorylation of AMPK (Fig. 3c).

### ME inhibits tumor growth in mouse oral orthotopic models

**Fig. 4:**
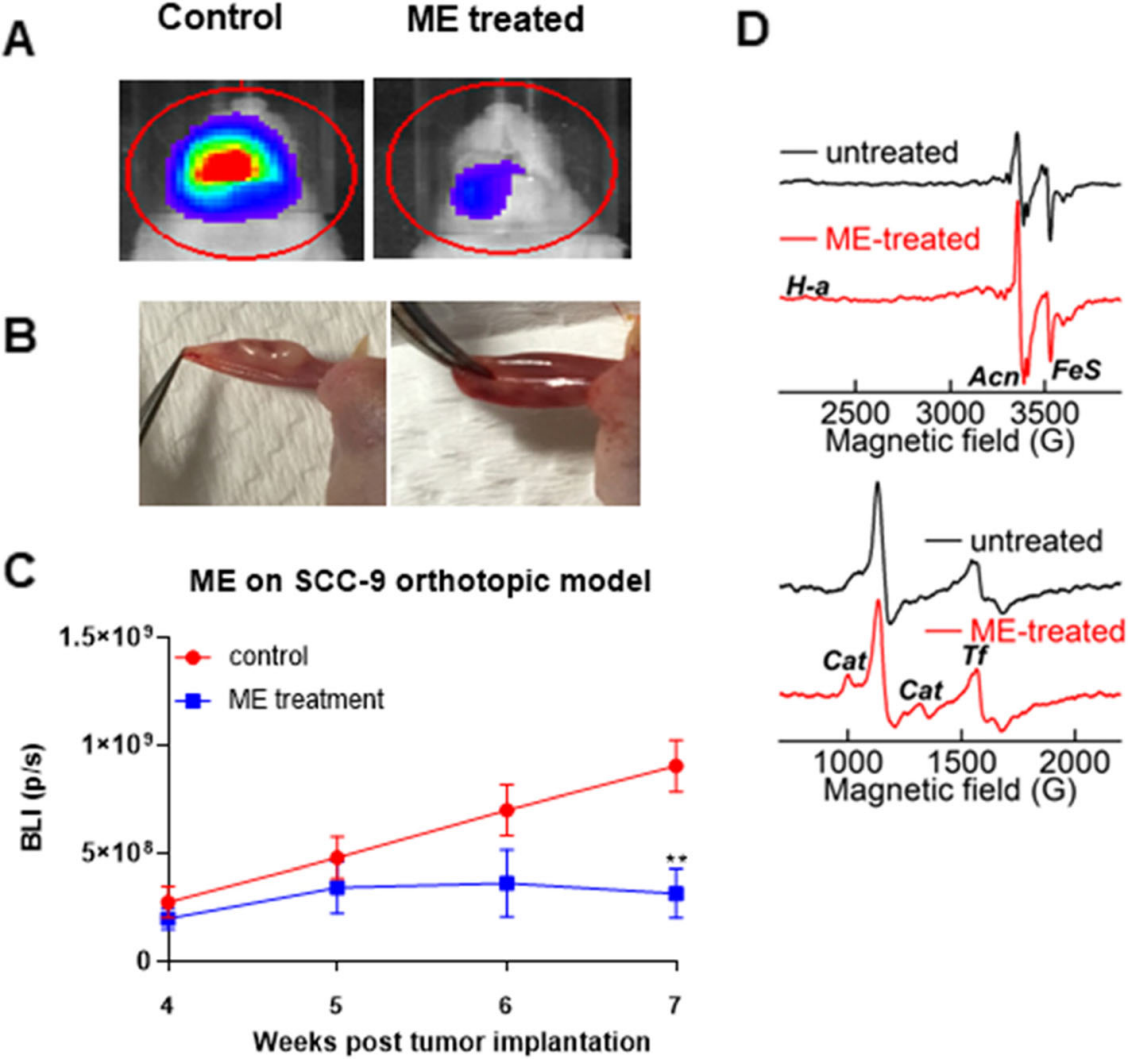
Inhibitory effect of ME on oral tumor growth in an orthotopic mouse model. **a** Representative BLI signal in control and ME -treated mice bearing orthotopic SCC-9 oral tumors which were treated with vehicle (corn oil), or ME (120 mg/kg, 5 times per week for 6 weeks starting 1 week after tumor inoculation). **b** Representative photographs of tumors from control and ME-treated mice. **c** BLI signal intensity of oral orthotopic SCC-9 tumors over time. **d** Effects of ME on EPR signals of oral orthotopic tumors harvested at the time of sacrifice.**P < 0.01

The efficacy of ME on oral tumor growth was evaluated using an orthotopic model of oral cancer in nude mice. SCC-9 cells were injected into the tongue of 6-week old mice. One week later, mice were treated with ME (120 mg/kg, oral administration 5 days per week) or vehicle (corn oil). During the treatment, the progression of tongue tumors was monitored by bioluminescence imaging (BLI). As shown in Fig. 4a-c, ME reduced BLI signals by ~ 72% as compared to control mice. Similar results were seen with a CAL 27 orthotopic model (Supplemental Figure S2).

### Effects of ME on EPR signals of oral orthotopic tumors

The effects of ME on mitochondrial ETC complexes and on promoting oxidative stress in CAL 27 oral tumors were determined by ex vivo low-temperature electron paramagnetic resonance (EPR). We have previously used this approach to measure changes in mitochondrial complexes in cell culture systems in vitro and in tissues ex vivo [31, 32]. Figure 4d shows EPR spectra of oral tumors from mice, either untreated or treated with ME. Signals highlighted are heme *a*_3_ of complex IV (*H-a*), catalase ferriheme (*Cat*), transferrin (*Tf*), aconitase (*Acn*) and iron-sulfur (*FeS*). The *H-a* of complex IV is EPR-silent in the reduced state and is only observed when present in the oxidized state. The strong signal at g = 1.94 (marked as *FeS*) is consistent with reduced forms of the [2Fe2S]^+^ and [4Fe4S]^+^ centers associated with mitochondrial complex I blockage. The ME-treated tumors also show a marked increase in the signal for oxidized *Acn*. Inactivation of aconitase occurs as a result of superoxide-induced oxidation of the [4Fe4S]^2+^ cluster (EPR silent) to a [3Fe4S]^+^ cluster (EPR active). The low-temperature EPR signal of the [3Fe4S]^+^ form of mitochondrial aconitase is distinctly different from that of cytosolic aconitase, exhibiting highly characteristic and distinctive absorptions in the g = 2.03 and 2.01 regions. The ME-treated tumors also show markedly enhanced signals for catalase (*Cat*) and transferrin (*Tf*). The enhanced *Cat* signal provides an additional marker for increased ROS, as it is overexpressed in response to peroxide. Increased *Tf* has been observed under many stress conditions and has been postulated to be a response to free iron. Overall, ME’s effects in oral tumors are clearly observable by EPR and include a sustained increase in ROS (oxidized Acn and increased Cat) and  in the redox potential experienced by FeS centers in complexes I & II.

### ME inhibits 4NQO-induced oral carcinogenesis

**Fig. 5:**
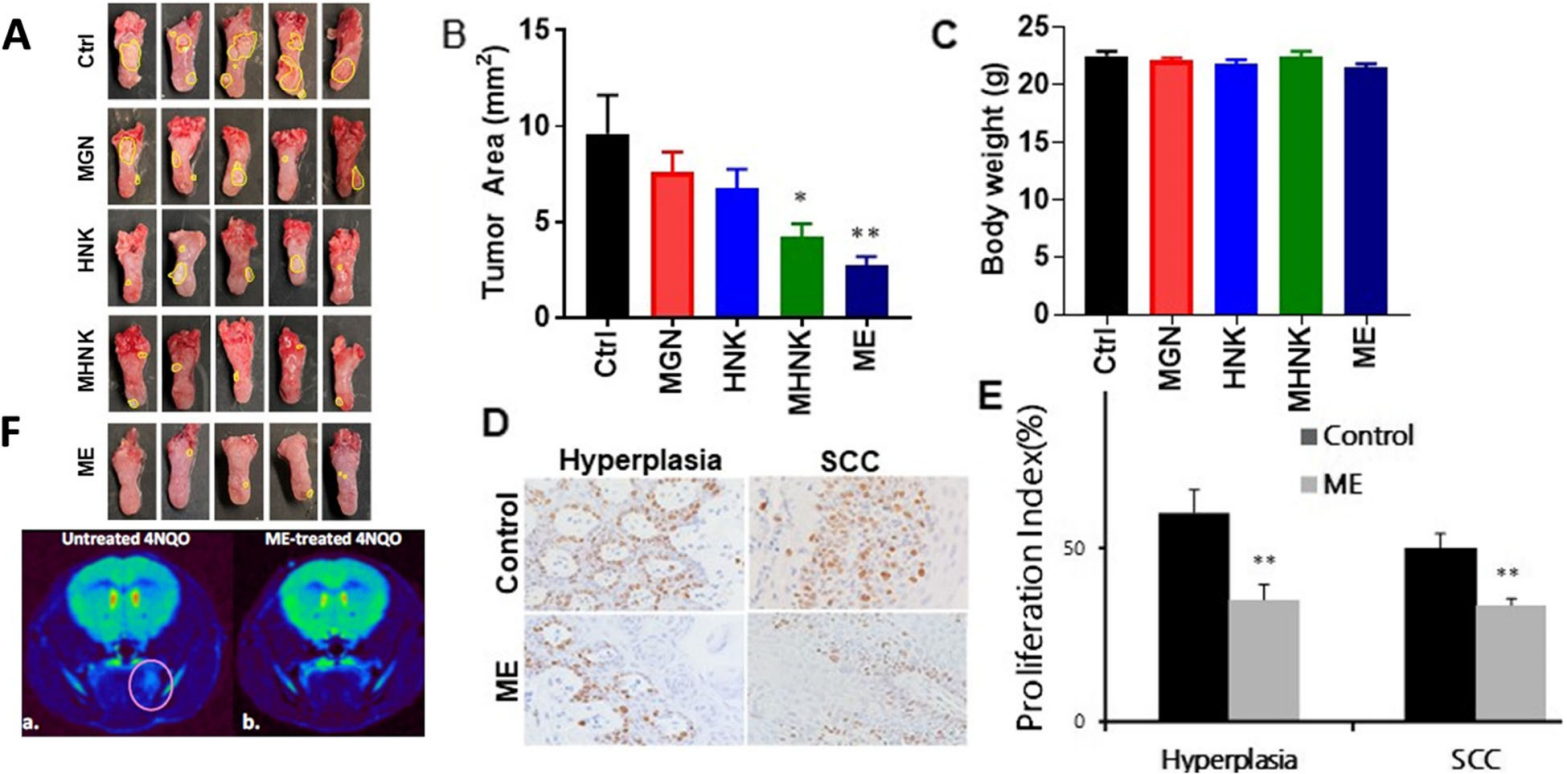
Inhibitory Effect of ME on 4NQO-induced oral cancer. **a** Representative images of 4NQO induced lesions. **b** Tumor area per mouse from 4NQO only (control) mice versus those treated with ME or individual agents from ME (*n* = 15). **c** Body weights of mice following the full duration of treatment with ME or its individual agents. **d** Representative IHC images for Ki-67. **e** Quantitation of Ki-67 from IHC analysis of animals. **f** Representative colorized T2w MRI scans of 4NQO mice (**a**) without and (**b**) with ME treatment. The lesion in the untreated mouse is encircled. *P < 0.05; **P < 0.01

The effects of ME and its active components on 4NQO-induced oral carcinogenesis were tested in C57BL/6 J mice. During the study, mice in the 4NQO only group showed no difference in body weight compared to those treated with ME or single compounds from ME (Fig. 5c*)*. Also, no significant differences in 4NQO consumption were observed between the mice in the control group (4-NQO only) and those treated with ME or single active agents from ME (data not shown). We observed mostly mild and severe dysplasias at 12 weeks after the start of the 4NQO-treatment, and SCCs after 20 weeks. The dysplastic areas showed disordered architecture, pronounced rete pegs, and cellular atypia characterized by increased nuclear size with few mitoses. Invasive squamous cell carcinomas were present in all mice at 20 weeks after the start of 4NQO treatment (Supplemental Figure S3). ME-treated mice showed significant inhibition of tumor lesions when compared with control mice or those treated with single compounds (Fig. 5a, b). Relative to control, ME significantly decreased the overall oral lesion area and the level of Ki67 (proliferation marker) (Fig. 5d, e). Perfusion MRI Methods have shown tremendous promise for the detection and evaluation of many solid tumors including those of the head and neck. Advanced MRI methods that monitor changes in tumor cell density and microvasculature were used to assess response to ME treatment. We obtained T2w MRI data in untreated (*n* = 7) and ME-treated (*n* = 6) 4NQO mice using the 9.4 T MRI. We observed lesion growth in the untreated mice but no lesion growth in ME-treated mice (Fig. 5f).

### ME targets STAT3, P70S6K, and HKII pathways

**Fig. 6:**
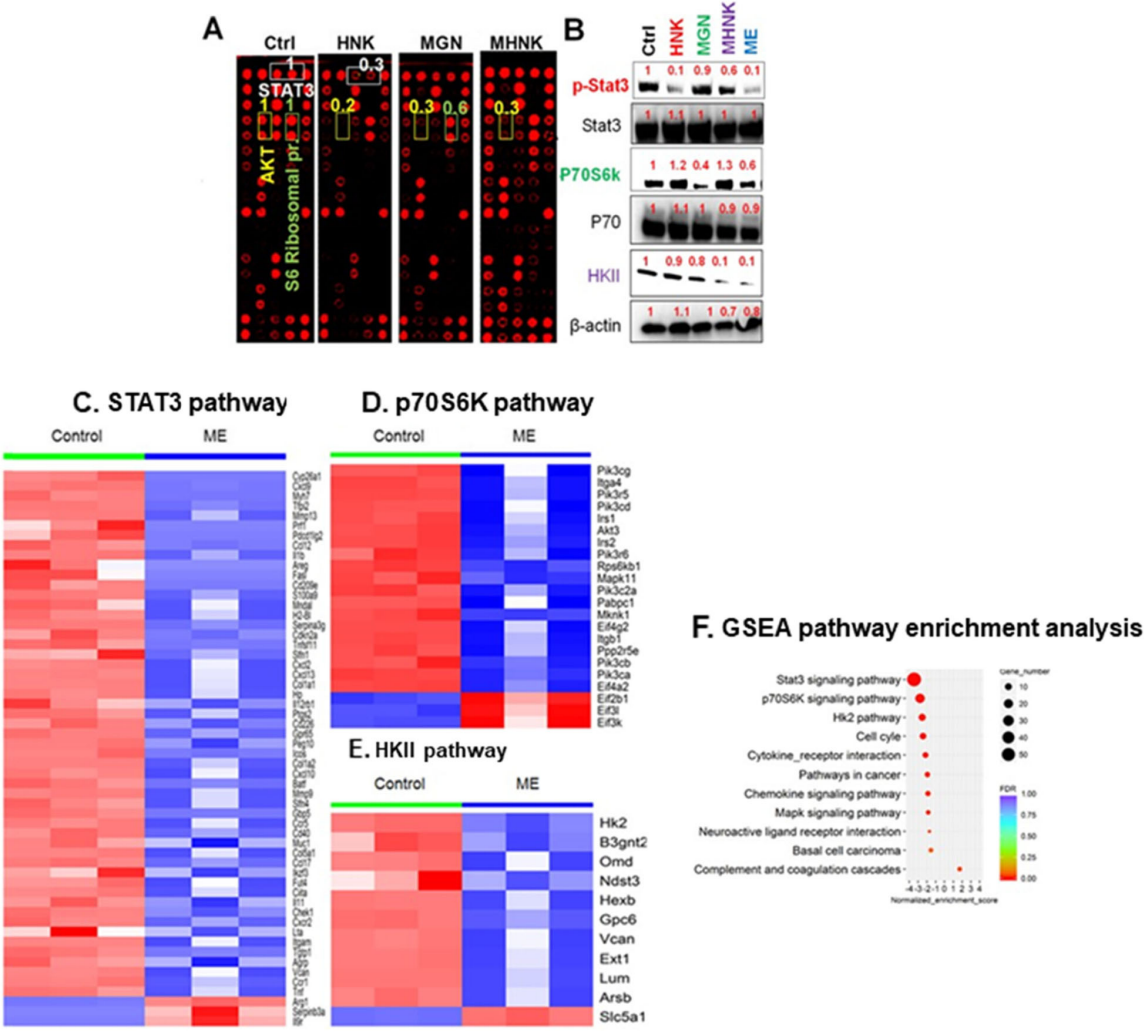
ME treatment changes STAT3, p70S6K, HKII and their associated pathways. **a** Receptor tyrosine kinase proteomic array of CAL 27 cells treated with HNK (20 μM), MGN (40 μM) or MHNK (20 μM). **b** Effects of HNK, MGN, MHNK, and ME on the phosphorylation status of STAT3 and p70S6K, and on the levels of HKII in CAL 27 cells. **c**-**e** ME treatment of oral tumors altered gene expression in the STAT3, p70S6K, and HKII pathways. **f** Pathway enrichment analysis identified the top 11 enriched pathways in ME-treated oral tumors vs. non-treated tumors, with STAT3, p70S6K and HKII pathways among the most enriched pathways

The mitochondrial effects of ME could affect various signaling events. Such potential mechanisms of ME and ME key components were examined via a receptor tyrosine kinase assay, which has been used extensively to study mechanisms of candidate cancer drugs [40]. This array (Fig. 6a) identified that, when used at concentrations close to their individual IC_50_ values, HNK, MGN, and MHNK affect different signaling events in oral cancer cells; these effects were validated via western blot (Fig. 6b). Specifically, HNK and MGN inhibited the phosphorylation of STAT3 and p70S6K, respectively, and MHNK decreased the levels of hexokinase II (HKII) (Fig. 6b). Interestingly, ME treatment decreased the levels of all three proteins, i.e. pSTAT3^Ser727^, p70S6K, and HKII (Fig. 6b Western blot, right lane).

The ability of ME to affect differential gene expression in oral tumors was determined. Using GSEA software [41], the top 11 enriched pathways were identified for ME-treated oral tumor samples vs. non-treated tumors. Interestingly, STAT3 signaling, p70S6K signaling and HKII pathways are among the most enriched pathways that are altered by ME, which agrees with the receptor tyrosine kinase array results (Fig. 6a, b). STAT3 signaling was the most represented pathway with 56 genes differentially expressed (53 downregulated, Fig. 6c), followed by p70S6K signaling with 22 genes differentially expressed (19 downregulated, Fig. 6d), and the HKII pathway with 11 genes differentially expressed (10 downregulated, Fig. 6e). These results confirm that these three molecular pathways (STAT3, p70S6K and HKII) were significantly down-regulated in the tumor samples of the ME group (Fig. 6f). All three pathways can regulate mitochondrial respiration and glycolysis.

**Fig. 7:**
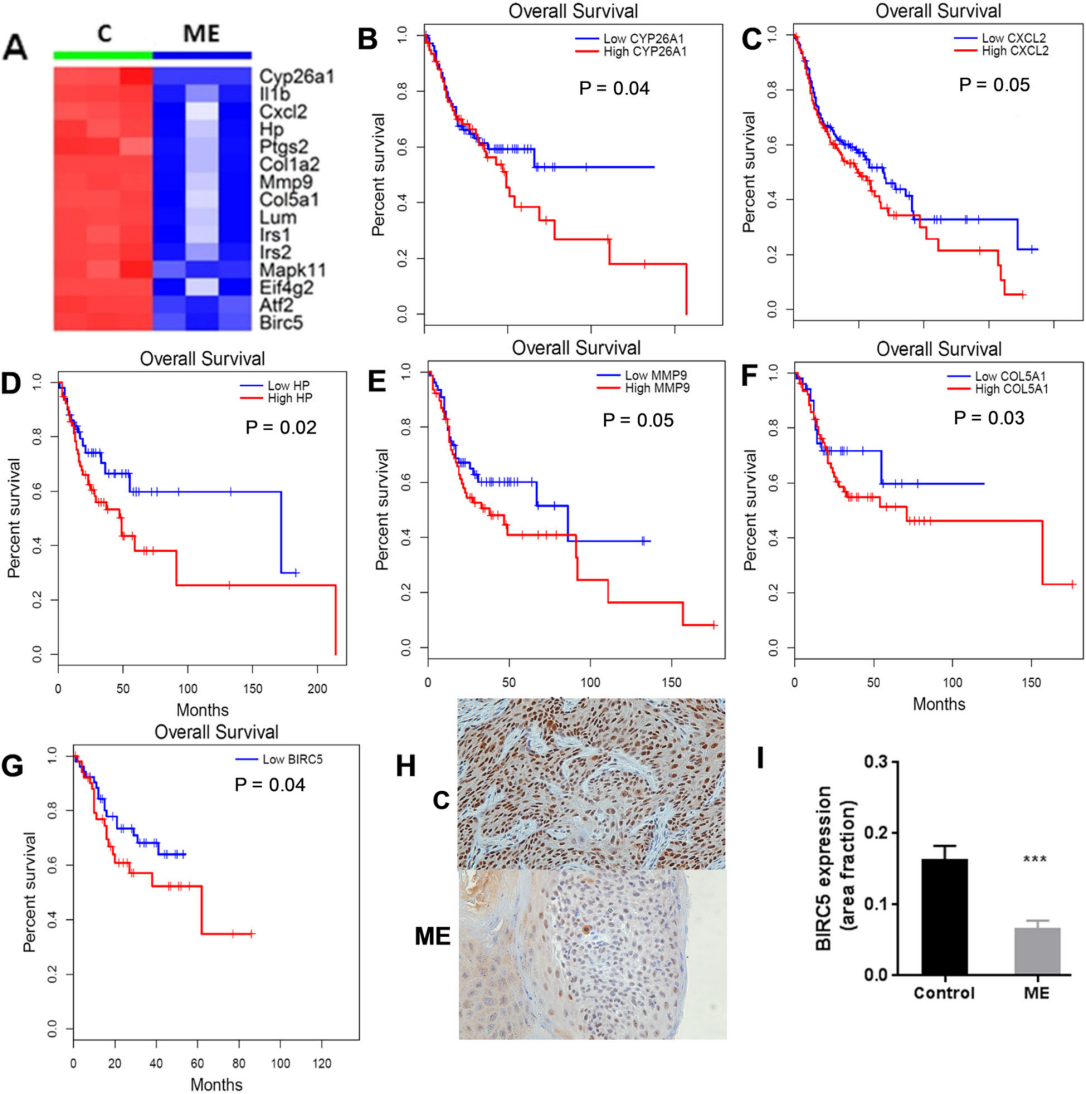
Identification of related genes by RNA-seq and their correlation with patient survival. **a** 15 selected genes representing ME-targeted pathways. **b**-**g** 6 representative genes predicted worse patient outcomes using TCGA cancer datasets with survival data. **h**, **i** Representative images and quantitation of BIRC5 expression in control and ME-treated mice (*n* = 6); ***P < 0.001

Using the genes from the top three pathways, including 23 STAT3, 3 HKII and 19 p70S6K pathway genes, we then screened these 45 genes for their potential to predict patient outcomes using TCGA cancer datasets in Head-Neck Squamous Cell Carcinoma (HNSC) patients (Fig. 7a-g). The number of genes we could screen was determined by the number of genes with available gene expression data in the TCGA database. A higher expression of 15 of these 45 genes was significantly associated with poor outcomes of cancer patients. As shown in Fig. 7, six of these representative genes predict worse outcomes according to survival data from the TCGA cancer datasets. Using IHC analysis, we found that ME treatment decreased the levels of BIRC5 (Fig. 7h, i), and since lower levels of BIRC5 predict better survival in patients (Fig. 7g), BIRC5 could serve as an in vivo biomarker indicating a beneficial response to ME.

### Pharmacokinetics of ME in animal tissues

The ME product we used is manufactured by SK Bioland. We conducted a detailed fingerprint analysis of this ME using mass spectrometry which identified at least 11 known compounds (Supplemental Fig S4), of which MHNK, HNK and MGN are the main components. We also used LC-MS/MS to determine the pharmacokinetics of MHNK, HNK and MGN in mice. Following oral administration of ME, all three compounds were broadly distributed with a large volume of distribution (Supplemental Fig S5). Elimination rates were moderate (half-lives of 2–3.5 h) for all three active compounds.

## Discussion

Chemoprevention is an important strategy for cancer. It can be categorized as either primary or secondary chemoprevention. For oral cancer, primary chemoprevention would be directed at patients with premalignant lesions (e.g. leukoplakia, erythroplakia). Secondary chemoprevention would be targeted at patients who have been treated for oral cancer with the goal to prevent recurrent disease (i.e. second primary tumors). ME has previously shown chemopreventive and antitumor efficacy against a variety of cancers. However, the relative content of its most abundant bioactive components (HNK, MGN, and MHNK) varies widely among various commercially available ME products (Supplementary Table S1), so specific products could have different anti-cancer potential. In this study, we tested a specific ME formulation (product 7, Supplemental Table S1) as a preventive agent for oral cancer. Relative to the other products listed in Table S1, this formulation has by far the highest content of MHNK, both on an absolute basis and relative to the contents of HNK and MGN. MHNK and HNK exhibited the lowest IC_50_ values for CAL 27 cells in vitro (Fig. 1d-f). MHNK (when tested as a sole agent) had the greatest effect on suppressing 4NQO-induced oral tumors in mice when compared with HNK and MGN (Fig. 5a, b). These data suggest that a relatively high content of MHNK within ME is important for enhancing the suppression of oral cancer. In addition to contributing to the inhibition of mitochondrial complex I, MHNK also markedly suppresses the glycolytic enzyme HKII which could render the cells less able to rely on glycolysis for energy generation.

Emerging cancer biology literature indicates a novel signaling and regulatory role for mitochondria-generated ROS in cancer cell proliferation, tumor growth, and metastasis [42]. The inhibition of mitochondrial complex I occurred within minutes of exposure to ME, suggesting that this is a key initial event for its effects on tumor cells. The strong EPR signal consistent with reduced forms of [2Fe2S]^+^ and 4[2Fe4S]^+^centers were observed in oral tumors of ME-treated mice (Fig. 4d) which provides in vivo evidence of mitochondrial complex I blockage. The inhibition of complex I is known to enhance mitochondrial oxidant generation. Consistent with this, ME resulted in peroxiredoxin oxidation (an endogenous indicator of excess H_2_O_2_ generation) (Fig. 3a). Tumors from ME-treated animals showed a marked increase in the signals for oxidized aconitase (which results from superoxide-induced damage) and for catalase (which is overexpressed in response to peroxide) (Fig. 4d). Thus, both in vitro and in vivo, there is substantial evidence that ME promotes sustained increases in ROS-mediated oxidative stress.

While the inhibition of complex I and the resulting ROS generation are early events in cancer cells exposed to ME, other subsequent events may ultimately contribute to the anti-proliferative effects. AMPK is a master regulator of cellular energy homeostasis and is typically activated in response to nutrient or energy deprivation. ME increased the activation (phosphorylation) of AMPK (Fig. 3c). The increased AMP/ATP ratio that we observed with ME treatment (Fig. 3b) is well known to promote AMPK activation [43, 44], although increased peroxide is another potential cause of AMPK activation [45]. AMPK activation ultimately leads to additional signaling events including decreased phosphorylation of the mTOR substrate p70S6K. Indeed, ME led to significantly decreased p70S6K phosphorylation in oral cancer cells. Constitutive activation of STAT3 in many tumors is important for tumor growth and progression [46]. ME suppressed STAT3 phosphorylation in oral cancer cells, and 53 genes associated with STAT3 signaling were suppressed in the tumors of ME-treated mice (Fig. 6). These pronounced effects on STAT3 could be important contributors to ME’s suppression of oral cancer. STAT3 is susceptible to oxidative inactivation [47, 48] and oxidized peroxiredoxins can oxidize STAT3 [39]. The Prx oxidation we observed is therefore a potential link between increased ROS and the inhibition of STAT3. Akt (protein kinase B) is another pro-survival factor that is activated in many cancers [49]. ME decreased the phosphorylation of Akt by ~ 50% in oral cancer cells (data not shown), and based on the RNA-seq, the expression of Akt3 was markedly suppressed in tumors from ME-treated mice (Fig. 6d). It is known that the Akt pathway regulates the expression of the glycolytic enzyme HKII [50] and we observed that ME markedly suppresses the levels of HKII (Fig. 6). Peroxiredoxin oxidation is another potential mechanism that can inhibit Akt signaling [51]. Our results therefore implicate potential links between the various effects of ME to inhibit oral cancer cells, including impaired complex I activity, increased ROS generation, Prx oxidation, activation of AMPK, inhibition of STAT3 and p70S6K phosphorylation, and suppression of HKII.

While HNK, MGN, and MHNK exhibited some efficacy for oral cancer when used alone, the anti-cancer effects were significantly enhanced when all three agents were combined or included as in the natural ME formulation (Fig. 1). Regarding the signaling events noted above, ME suppressed the levels of HKII and the phosphorylation of STAT3 and p70S6K (Fig. 6). When tested as sole agents, each active ingredient primarily affected different signaling molecules. HNK mainly decreased the phosphorylation of STAT3, MGN decreased the phosphorylation of p70S6K, and MHNK decreased the levels of HKII (Fig. 6). RNA-seq of tumors from ME-treated animals confirmed that these three signaling pathways were down-regulated by ME. More importantly, key genes downregulated by ME were found to be reversely correlated with patients’ survival (Fig. 7), suggesting that ME treatment may present clinical benefits to oral cancer patients.

## Conclusion

We conclude that a core proximal underlying mechanism of action for ME is its ability to inhibit mitochondrial bioenergetics. We demonstrated that ME suppresses mitochondrial respiration and increases ROS generation which leads to Prx oxidation, AMPK activation, and the inhibition of STAT3 in oral cancer cells. Using low-temperature EPR to analyze mouse orthotopic oral tumors, we confirmed that complex I inhibition, increased ROS production and ROS-mediated oxidative stress are associated with ME’s ability to suppress oral tumors. These findings provide key new insights into the chemopreventive mechanisms and potential of ME as a safe and effective agent to prevent oral cancer.

## Supplementary information

**Additional file 1: Table S1.** Variability in active compounds of magnolol extracts in various marketed products of ME found in local pharmacy or popular internet sites. Product #7 is the one we called ME.

**Additional file 2: Figure S1.** Experimental design for studies on the inhibitory effect of ME on tumor development in 4NQO-induced oral cancer mouse.

**Additional file 3: Figure S2.** ME inhibits tumor growth in Cal-27 oral orthotopic models

**Additional file 4: Figure S3.** Histopathology of oral lesions. Top panel: 12 weeks after starting the 4NQO treatment, dysplastic changes are observed; Bottom panel: 20 weeks after starting the 4NQO treatment, invasive SCC are observed’.

**Additional file 5: Figure S4.** A. HPLC fingerprint of ME used in the current study (made by SK Bioland). B Structures of 11 compounds identified in the used ME, from LC-MS/MS analyses.

**Additional file 6: Figure S5.** The pharmacokinetics study of the three active components of ME. (a) HPLC chromatogram of ME. (b-d) pharmacokinetic profiles of HNK, MGN and MHNK in mouse tongue (b), oral cavity (c) and blood (d).

## Data Availability

Not applicable.

## Abbreviations

Acn: Aconitase
BEGM: Bronchial epithelial growth medium
Cat: Catalase ferriheme
DMSO: Dimethyl sulfoxide
EPR: Electron paramagnetic resonance
FeS: Iron-sulfur
HNK: Honokiol
HNSC: Head-Neck Squamous Cell Carcinoma
ME: Magnolia extract
MHNK: 4-O-methylhonokiol
MGN: Magnolol
NEM: *N*-ethylmaleimide
NHBE: Normal human bronchial epithelial cells
PMP: Plasma Membrane Permeabilizer
Prxs: Peroxiredoxins
RNA-seq: RNA sequencing
ROS: Reactive oxygen species
SCC: Squamous cell carcinoma
SDS-PAGE: Sodium dodecyl sulfate polyacrylamide gel electrophoresis
STAT3: Signal transducer and activator of transcription
Tf: Transferrin
