# Supplementary material for: Magnolia extract is effective for the chemoprevention of oral cancer through its ability to inhibit mitochondrial respiration at complex I
Source: Cell Commun Signal. 2020 Apr 7;18:58. doi: 10.1186/s12964-020-0524-2 (PMC7140380; doi:10.1186/s12964-020-0524-2)
Supplement: Supplementary file 2 — Additional file 1: Table S1. Variability in active compounds of magnolol extracts in various marketed products of ME found in local pharmacy or popular internet sites. Product #7 is the one we called ME. [file 12964_2020_524_MOESM1_ESM.docx]

**Supplemental Table s1.** Variability in active compounds of magnolol extracts in various marketed products of ME found in local pharmacy or popular internet sites. Product #7 is the one we called ME.
