# Supplementary material for: Magnolia extract is effective for the chemoprevention of oral cancer through its ability to inhibit mitochondrial respiration at complex I
Source: Cell Commun Signal. 2020 Apr 7;18:58. doi: 10.1186/s12964-020-0524-2 (PMC7140380; doi:10.1186/s12964-020-0524-2)
Supplement: Supplementary file 3 — Additional file 2: Figure S1. Experimental design for studies on the inhibitory effect of ME on tumor development in 4NQO-induced oral cancer mouse. [file 12964_2020_524_MOESM2_ESM.docx]

**Supplemental fig s1: Experimental design for Inhibitory Effect of ME on 4NQO-induced oral cancer mouse model**
