# Supplementary material for: Magnolia extract is effective for the chemoprevention of oral cancer through its ability to inhibit mitochondrial respiration at complex I
Source: Cell Commun Signal. 2020 Apr 7;18:58. doi: 10.1186/s12964-020-0524-2 (PMC7140380; doi:10.1186/s12964-020-0524-2)
Supplement: Supplementary file 4 — Additional file 3: Figure S2. ME inhibits tumor growth in Cal-27 oral orthotopic models [file 12964_2020_524_MOESM3_ESM.docx]

**Supplemental fig s2: ME inhibits tumor growth in Cal-27 oral orthotopic models**
