# Supplementary material for: Magnolia extract is effective for the chemoprevention of oral cancer through its ability to inhibit mitochondrial respiration at complex I
Source: Cell Commun Signal. 2020 Apr 7;18:58. doi: 10.1186/s12964-020-0524-2 (PMC7140380; doi:10.1186/s12964-020-0524-2)
Supplement: Supplementary file 5 — Additional file 4: Figure S3. Histopathology of oral lesions. Top panel: 12 weeks after starting the 4NQO treatment, dysplastic changes are observed; Bottom panel: 20 weeks after starting the 4NQO treatment, invasive SCC are observed’. [file 12964_2020_524_MOESM4_ESM.docx]

**Supplemental fig s3: Histopathology of oral lesions.** Top panel: 12 weeks after starting the 4NQO show dysplastic changes; Bottom panel: 20 weeks after starting the 4NQO show invasive SCC
