# Supplementary material for: Magnolia extract is effective for the chemoprevention of oral cancer through its ability to inhibit mitochondrial respiration at complex I
Source: Cell Commun Signal. 2020 Apr 7;18:58. doi: 10.1186/s12964-020-0524-2 (PMC7140380; doi:10.1186/s12964-020-0524-2)
Supplement: Supplementary file 6 — Additional file 5: Figure S4. A. HPLC fingerprint of ME used in the current study (made by SK Bioland). B Structures of 11 compounds identified in the used ME, from LC-MS/MS analyses. [file 12964_2020_524_MOESM5_ESM.docx]

**Supplemental fig s4: A**. HPLC fingerprint of ME to be used in the current study. **B** Structures of 11 identified compounds as defined by LCMS/MS with high certainty from ME made by SK Bioland.
