# Supplementary material for: Magnolia extract is effective for the chemoprevention of oral cancer through its ability to inhibit mitochondrial respiration at complex I
Source: Cell Commun Signal. 2020 Apr 7;18:58. doi: 10.1186/s12964-020-0524-2 (PMC7140380; doi:10.1186/s12964-020-0524-2)
Supplement: Supplementary file 7 — Additional file 6: Figure S5. The pharmacokinetics study of the three active components of ME. (a) HPLC chromatogram of ME. (b-d) pharmacokinetic profiles of HNK, MGN and MHNK in mouse tongue (b), oral cavity (c) and blood (d). [file 12964_2020_524_MOESM6_ESM.docx]

**Supplemental fig s5: The pharmacokinetics study of three compounds**
